# Prevalence of anhedonia, anxiety, and their impact on food consumption among postgraduate Qassim University students

**DOI:** 10.3389/fnut.2024.1445125

**Published:** 2024-10-31

**Authors:** Sarah Alrehaili, Abeer A. Afifi, Reham M. Algheshairy, Taqwa Bushnaq, Talal Ali F. Alharbi, Hend F. Alharbi

**Affiliations:** ^1^Department of Food Science and Human Nutrition, College of Agriculture and Food, Qassim University, Buraydah, Saudi Arabia; ^2^Department of Nutrition, Buraidah Central Hospital, Qassim Health Cluster, Ministry of Health, Buraydah, Saudi Arabia; ^3^Nutrition and Food Science Department, Food Industries and Nutrition Institute, National Research Centre, Dokki, Cairo, Egypt; ^4^Department of Food Science and Nutrition, College of Science, Taif University, Taif, Saudi Arabia; ^5^Department of Psychiatric, Mental Health and Community Health Nursing, College of Nursing, Qassim University, Buraidah, Saudi Arabia

**Keywords:** diet, food intake, dietary intake, nutrition, anhedonia, anxiety, health

## Abstract

**Objectives:**

A prevalent symptom of many mental health issues, such as depression, is anhedonia, which is the inability to feel joy or pleasure. Tension-induced sensations, anxious thoughts, and bodily alterations are the hallmarks of anxiety. One known environmental factor that affects mental health is diet. In this implication, eating appetizing meals has been proposed to reduce unpleasant feelings like worry. This study aimed to indicate whether eating habits among postgraduate students are related to symptoms of anhedonia and/or anxiety.

**Methods:**

In a cross-sectional study, responses were collected from a total of 393 postgraduate students. Each student self-answered the General Anxiety Disorder-7, Snaith-Hamilton Pleasure Scale for Anhedonia, and the Food Consumption Questionnaire and reported the body weight.

**Results:**

The prevalence of anhedonia was 28%, moderate anxiety and severe anxiety were 15.7 and 10.9%, respectively. In this research, results showed that sugary foods, fast food, and fried foods were positively associated with anxiety and anhedonia. However, fruits and vegetables were negatively associated with anxiety and anhedonia. Linear regression showed that fruit and drinking water consumption was significant with the number of meals/day. There was no significance between the number of meals per day and anhedonia; however, there was statistical significance with the total GAD-7 scale score regarding the number of meals/per day.

**Conclusion:**

Anhedonia and anxiety are associated with food consumption, especially foods rich in sugars, fast and fried, which help to increase positive feelings. Future studies should focus on understanding the relationship between food consumption and following a healthy diet and improving and reducing symptoms of anxiety and anhedonia in those who suffer from a stressful or task-filled lifestyle, such as students, especially postgraduate students. In addition, it focuses on the importance of awareness of the symptoms of anhedonia, which is classified as a depressive disease, and how to control anxiety to maintain better mental health.

## 1 Introduction

Anhedonia is a primary symptom of many different types of mental illnesses and neurological conditions, including eating disorders, Parkinson’s disease, severe depression, schizophrenia, and mild anxiety (1). Understanding the symptoms of mental health disorders and obesity as comorbidities is becoming increasingly important due to the rising global prevalence of both conditions. Anhedonia is one of the essential symptoms in the definitions of depression, various mood disorders, and schizophrenia (1–3). Thus, anhedonia is also included in the official diagnostic instruments for Major Depressive Disorder (MDD), such as the Major Depressive Inventory (4) and the Diagnostic and Statistical Manual of Mental Disorders, Fifth Edition (DSM-5) (5). Self-report measures such as the Snaith-Hamilton Pleasure Scale (SHAPS) (6) and the Chapman Physical and Social Anhedonia Scales (7) have confirmed the link between anhedonia and these disorders, as have behavioral assessments in human studies with MDD and schizophrenia patients, as well as neuroscientific experimental animal studies (1, 3, 8). Similar to MDD, anxiety disorders are a subset of mental illnesses that have also been linked to anhedonic characteristics, albeit not to the same extent. Anhedonia has been proposed as the mediating factor between MDD and anxiety, which is often considered to be the former’s precursor (9, 10). Positive reward experiences may be purposefully avoided by those who suffer from anxiety, which could eventually lead to depressed symptoms as well (9, 10). Anhedonia is widespread in people with Alzheimer’s disease, Parkinson’s disease, eating disorders, and substance use disorders, in addition to the well-established links to depression, schizophrenia, and other mood disorders (1).

Diet is thought to be an environmental variable that has a significant impact on mental health (11). Dietary aspects and psychological health research have yielded mixed results, and there is little evidence of the association between food consumption and mental health in constrained situations. Several research studies have proposed that eating palatable foods (high-calorie foods high in sugar, other carbohydrates, and/or fat) is a strategy to reduce negative emotions like anxiety that can be brought on by stressors (12, 13). According to studies by Kim and others (14, 15), these stressors may also cause many people to choose different foods, which could increase their calorie intake. Anxiety among medical students ranges between 20 and 60%; academic stress, often linked to anxiety, is related to poor eating habits among medical students, including skipping breakfast, consuming unhealthy foods, and insufficient fruit and water intake, emphasizing the need for stress management and healthy eating habits (16). Twenty-one systematic review studies found that anhedonia was significantly higher in eating disorder groups compared to healthy controls; however, there is no significant difference found between diagnostic groups (17). Anhedonia may be correlated with increased eating disorder symptom severity.

Similarly, anhedonia, or the reduced capacity for pleasure, is linked to a preference for appetizing foods and may play a more significant role than physiological cues like hunger and fullness (18). Hedonistic hunger is the term for this phenomenon, which is a psychological element based on traits. No physiological hunger is characterized by an excessive sensitivity to reward, pleasure, and food desire (19). Furthermore, research has linked it to a number of detrimental health outcomes, such as obesity and maladaptive eating patterns (such as binge eating, unhealthy snacking, and the absence of hunger) (19). A study reveals a link between anhedonic traits, depression, anxiety and food pleasure perception. People with depression and anxiety experience reduced pleasure from food aspects, while eating alone increases pleasure. These individuals prefer sensory elements of food (20).

The prevalence of anxiety among university students suggests that it is a common issue that may influence their eating behaviors and overall health. According to a study, emotional eating is linked to anxiety in 51.3% of female university students, suggesting that anxiety may cause these behaviors. It is essential to address high levels of stress and anxiety because they can result in unhealthy eating habits and weight gain (21). This study evaluated anxiety using the General Anxiety Disorder-7 (GAD-7) questionnaire (22), a tool commonly used in studies. Anhedonia was assessed using the method SHAPS validated in Arabic (6, 23). SHAPS, with its strong psychometric properties, convergence validity, cultural adaptation, broad applicability, and focus on core symptoms of mental health disorders such as schizophrenia and Major Depressive Disorder, is a valid tool for assessing anhedonia (23). Therefore, this study aims to demonstrate the prevalence of anhedonia and anxiety and its effects on dietary consumption among postgraduate students at Qassim University. To the best of our knowledge, with the lack of results on anhedonia, especially in Saudi Arabia, this study expands the data on anhedonia and anxiety, its prevalence and its general effect on dietary habits or consumption among postgraduate students.

## 2 Materials and methods

### 2.1 Study design and participants

This observational cross-sectional study was carried out among postgraduate Qassim University students in Saudi Arabia. The study was done in accordance with the Declaration of Helsinki and was approved by Qassim University’s Institutional Review Board (or Ethics Committee) No. 08-36-23, issued on 3 May 2023. It created an online questionnaire using Google Forms (Google LLC, Menlo Park, CA, USA). The questionnaire was invited to be completed through postgraduate student groups and emails. The selection criteria were the following: both genders, aged between 22 and 55 years old (24–26). The exclusion criteria were pregnant, postnatal or breastfeeding, and those who were receiving pharmacological or psychological treatments for depression, anxiety disorders, stress, or mood disorders (27, 28). Subjects with diseases requiring nutritional intervention were also excluded. A first screening questionnaire includes questions to identify exclusion. If a participant answered “YES” to any of the screening questions, the questionnaire immediately closed, and participation ended. Twenty postgraduate students were excluded based on the exclusion criteria or because of incomplete or random answers.

All 393 postgraduate students expressed interest in volunteering to participate in the study. The questionnaire included six sections; the average completion time was 15 min. Before starting the questionnaire, each participant had to evaluate the study’s aims and provide electronic informed consent in section one. The researcher explained the questionnaire’s content and answered any questions if there were any inquiries. Each postgraduate student was informed of the possibility of withdrawing from participation at any time. Raosoft calculated the sample size online to determine the respondents’ requirements with an error margin to achieve the appropriate confidence level. To attain a 95% confidence level and a 3.15% margin of error, a minimum sample size of 385 is required to meet the study objectives.

### 2.2 Questionnaire

The self-administration questionnaire was used. Experts in related fields reviewed the questionnaire. Additionally, the questionnaire’s external reviewers provided feedback and ideas for development. Based on their published research and experience in human nutrition, the experts chose and forged connections among themselves. We implemented a number of modifications to the questionnaire to enhance its validity, reliability, and scientific value in data collection. In order to confirm the validity and reliability of the questionnaire, pilot research with 30 participants was carried out. After being obtained, Cronbach’s alpha was found to be outstanding, surpassing 80% for parameters that were measured. The questionnaire contains six sections: (1) the aims and consent form, (2) demographic information, (3) the food consumption, (4) health and lifestyle information, (5) the General Anxiety Disorder-7 (GAD-7) and (6) the Snaith-Hamilton Pleasure Scale for Anhedonia (SHAPS).

#### 2.2.1 Demographic information

The demographic information included age, marital status, number of family members, university department, postgraduate education level, monthly income, number of children and area of residence for all student participants.

#### 2.2.2 Food consumption

Food consumption was assessed using food frequency questions from Linker’s Choices (29): consumption of sugary drinks, fried foods, pastries, consumption of soda drinks, and consumption of vegetables and fruits was assessed during the week (28).

#### 2.2.3 Health and lifestyle information

Lifestyle information focused on smoking status, being infected with the coronavirus, physical activity practice, sleeping hours per day, whether contraceptive pills are taken and suffering chronic diseases. Participants reported their height in cm and weight in kg, which were used to calculate their Body Mass Index (BMI) (kg/m^2^) (30).

#### 2.2.4 General Anxiety Disorder-7 (GAD-7)

The Arabic version of GAD-7 is used to evaluate Anxiety symptoms (31, 32). The GAD-7 is a valid tool to assess physiological (somatic) and cognitive aspects of anxiety in the general population that the subject may have experienced during the last 2 weeks before its application (33). The questionnaire consists of seven questions. The scouring is as follows: zero (not at all), one (several days), two (more than half of the days), and three (nearly every day). The GAD-7 score ranges from 0 to 21, with the higher scores indicating more anxiety (34). The cutoff values for mild, moderate, and severe anxiety symptoms are 5, 10, and 15, respectively (35).

#### 2.2.5 The Snaith-Hamilton Pleasure Scale for Anhedonia (SHAPS)

The Arabic version of SHAPS is used to assess anhedonia (23). This scale considers 14 items with four-point Likert scales from which the participants must choose the one that best describes the situation: totally disagree, disagree, agree, totally agree. Any “Agree” response is rated 0; any “Disagree” response is scored 1. The classification of anhedonic was used in the original scoring of SHAPS (score > 2) (6, 36, 37).

### 2.3 Statistical analysis

The SPSS (V.26.0 IBM, Armonk, NY, USA) and Excel were used to analyze data and the frequency distributions. Percentages were used to summarize categorical variables. A Chi-squared was used to compare the variables, including self-reported food consumption, anxiety (minimal/mild/moderate/severe), and anhedonia (normal/abnormal). BMI and body weight (mean, standard deviation) were analyzed using a T-test and one-way ANOVA. A linear regression was analyzed on the dependent variable. Statistical significance was stated as a *p*-value of less than 0.05.

## 3 Results

### 3.1 Demographic characteristics

Table 1 shows the frequency of demographic characteristics of the study population. A total of 393 participants consisted of females and males, mostly aged between 22 and 35 years old, at the rate of 77.6% of the total sample. 96.2% of the total participants are Saudi Ariba of Nationality. Female participants are the most active participants in this study at the rate of 79.1%. 91.3% of the participants live in cities, which showed that more than half of the sample, at a rate of 60.8%, 239 female and male participants, live in Buryadah city.

**Table 1:** The frequency of demographic characteristics of the study population.

Variables	Items	Total sample	
		*N*	%
		393	100
Age	22–35	305	77.6%
	36–45	84	21.4%
	≧46	4	1%
Nationality	Saudi	378	96.2%
	Non-Saudi	15	3.8%
Gender	Female	311	79.1%
	Male	82	20.9%
Collage	College of Dentistry	1	0.3%
	College of Applied Medical Sciences	2	0.5%
	College of Pharmacy	1	0.3%
	College of Computer	12	3.1%
	College of Medicine	1	0.3%
	College of Business	39	9.9%
	College of Health Sciences	7	1.8%
	College of Education	100	25.4%
	College of Science and Literature	104	26.5%
	College of Designs	12	3.1%
	College of Agriculture and Food	59	15%
	College of Sharia and Islamic Studies	55	14%
Place of living	Urban	359	91.3%
	Rural	34	8.7%
Area of residence	Buraydah	239	60.8%
	Unaizah	8	2%
	Ar Rass	2	0.5%
	Al Mithnab	3	0.8%
	AlBukayriyah	5	1.3%
	AlBadayea	42	10.7%
	AlAsyah	32	8.1%
	AlNabhaniyah	7	1.8%
	Uyun AlJiwa	16	4.1%
	Riyadh AlKhabra	18	4.6%
	AlShimasiyah	4	1%
	Uqalt as-Suqur	5	1.3%
	Daria	12	3.1%

Data presented as *n* (%) for categorical variables.

### 3.2 The associations between anhedonia and anxiety with demographics

Table 2 shows the relationship of anhedonia and anxiety with demographics. The mild anxiety level is more common among the ages of 22 up to 35 years, female students, and married participants, with a moral significance of less than 0.05. Participation in Category 35–35 Was the highest, where the percentage of abnormal anhedonia was 30.2%. Normal anhedonia in females is 73.6%, and in males, it is 65.9%. There was a moral significance about marital status, as the abnormal anhedonia ratio was 33.7, 22.8, and 18.8% for singles, married and divorced, respectively. In terms of level of education, the number of students who are in the research phase suffer from Mild levels of anxiety is 76. In contrast, 14 students reach the level of severe anxiety. There was a high level of abnormal anhedonia among students at the 4th level, at 46.5%.

**Table 2:** The associations of demographic characteristics of the study population.

Variables	Total	SHAPS		*p*-value	GAD-7				*p*-value
		Normal	Abnormal		Minimal	Mild	Moderate	Severe	
Age									
22–35	305 (77.6)	213 (69.8)	92 (30.2)	0.198	88 (22.4)	128 (32.6)	56 (14.2)	33 (8.4)	0.030
36–45	84 (21.4)	67 (79.8)	17 (20.2)		17 (4.3)	51 (13)	6 (1.5)	10 (2.5)	
≧46	4 (1)	3 (75)	1 (25)		2 (0.5)	2 (0.5)	0.0 (0)	0.0 (0)	
Gender									
Female	311 (79.1)	229 (73.6)	82 (26.4)	0.163	70 (17.8)	150 (38.2)	54 (13.7)	37 (9.4)	0.001
Male	82 (20.9)	54 (65.9)	28 (34.1)		37 (9.4)	31 (7.9)	8 (2)	6 (1.5)	
Level of education									
1st level	67 (17)	49 (73.1)	18 (26.9)	0.104	27 (6.9)	31 (7.9)	4 (1)	5 (1.3)	0.035
2nd level	13 (3.3)	9 (69.2)	4 (30.8)		6 (1.5)	3 (0.8)	2 (0.5)	2 (0.5)	
3rd level	58 (14.8)	45 (77.6)	13 (22.4)		11 (2.8)	24 (6.1)	14 (3.6)	9 (2.3)	
4th level	43 (10.9)	23 (53.5)	20 (46.5)		10 (2.5)	19 (4.8)	5 (1.3)	9 (2.3)	
Conducting the research	155 (39.4)	113 (72.9)	42 (27.1)		36 (9.2)	76 (19.3)	29 (7.4)	14 (3.6)	
Other (writing up, VIVA, etc.)	57 (14.5)	44 (77.2)	13 (22.8)		17 (4.3)	28 (7.1)	8 (2)	4 (1)	
Marital status									
Single	193 (49.1)	128 (66.3)	65 (33.7)	0.045	55 (14)	71 (18.1)	38 (9.7)	29 (7.4)	0.010
Married	184 (46.8)	142 (77.2)	42 (22.8)		48 (12.2)	101 (25.7)	22 (5.6)	13 (3.3)	
Divorced	16 (4.1)	13 (81.3)	3 (18.8)		4 (1)	9 (2.3)	2 (0.5)	1 (0.3)	
Monthly income (SR)									
<5,000	53 (13.5)	32 (60.4)	21 (39.6)	0.123	14 (3.6)	27 (6.9)	6 (1.5)	6 (1.5)	0.229
5,000–10,000	124 (31.6)	92 (74.2)	32 (25.8)		33 (8.4)	55 (14)	19 (4.8)	17 (4.3)	
10,000–15,000	109 (27.7)	76 (69.7)	33 (30.3)		21 (5.3)	53 (13.5)	22 (5.6)	13 (3.3)	
>15,000	107 (27.2)	83 (77.6)	24 (22.4)		39 (9.9)	46 (11.7)	15 (3.8)	7 (1.8)	
No. family member									
2–4	148 (37.7)	106 (71.6)	42 (28.4)	0.528	34 (8.7)	83 (21.1)	16 (4.1)	15 (3.8)	0.012
5–7	156 (39.7)	113 (72.4)	43 (27.6)		40 (10.2)	70 (17.8)	27 (6.9)	19 (12.2)	
8–10	69 (17.6)	47 (68.1)	22 (31.9)		27 (6.9)	18 (4.6)	15 (3.8)	9 (2.3)	
More than 10	20 (5.1)	17 (85)	3 (15)		6 (1.5)	10 (2.5)	4 (1)	0.0 (0)	
No. children									
One child	75 (14.5)	37 (64.9)	20 (35.1)	0.027	15 (3.8)	25 (6.4)	12 (3.1)	5 (1.3)	0.115
Two children	63 (16)	48 (76.2)	15 (23.8)		16 (4.1)	34 (8.7)	8 (2)	5 (1.3)	
Three to four children	89 (22.6)	72 (80.9)	17 (19.1)		23 (5.9)	46 (11.7)	11 (2.8)	9 (2.3)	
Five and more	27 (6.9)	23 (85.2)	4 (14.8)		15 (3.8)	7 (1.8)	3 (0.8)	2 (0.5)	
Non	157 (39.9)	103 (65.6)	54 (34.4)		38 (9.7)	69 (17.6)	28 (7.1)	22 (5.6)	
Type home of living									
Rental flat	114 (29)	85 (74.6)	29 (25.4)	0.458	26 (6.6)	59 (15)	17 (4.3)	12 (3.1)	0.033
Owner flat	72 (18.3)	50 (69.4)	22 (30.6)		22 (5.6)	32 (8.1)	12 (3.1)	6 (1.5)	
Villa	197 (50.1)	143 (72.6)	54 (27.4)		56 (14.2)	89 (22.6)	32 (8.1)	20 (5.1)	
Student accommodation	7 (1.8)	4 (57.1)	3 (42.9)		2 (0.5)	1 (0.3)	0.0 (0)	4 (1)	
Military housing unit	3 (0.8)	1 (33.3)	2 (66.7)		1 (0.3)	0.0 (0)	1 (0.3)	1 (0.3)	

Data presented as *n* (%) for categorical variables; *p*-value < 0.05 considered significant.

The highest normal anhedonia was for those who lived in an owner’s flat, as the normal percentage was 74.6%, and abnormal anhedonia was the high percentage for those who lived in Military housing units, 66.7%. There appeared to be a drop in anxiety levels among participants whose family members consisted of more than 10 persons. In comparison, anxiety levels increased among those whose family members consisted of 5 up to 7 persons, reaching a mild level of anxiety at the rate of (*n* 70, at 17.8%) followed by a severe level of anxiety at the rate of (*n* 19, at 12.2%). The results showed that families of 8–10 people have higher abnormal anhedonia levels at 31.9%, while the normal anhedonia rate increases in families of more than 10 people at 85%. Participants who do not have children have normal anhedonia at 65.6%. The result was similar for those with one child, reaching 64.9%, with a significance of 0.027.

The highest percentage of normal anhedonia was for those whose monthly income was more than 15,000 riyals, about 77.6%, while the highest percentage of abnormal anhedonia was among participants with a monthly income of less than 5,000 per month, where the percentage 39.6%.

### 3.3 The associations between anhedonia and anxiety with food consumption

Table 3 shows the association between anhedonia and anxiety levels with food consumption. It has been noticed that the level of severe anxiety increased among participants who intake two main meals a day at 7.1%. The results showed the highest percentage of normal anhedonia among those who ate three meals, at 76.8%, while the highest rate of abnormal anhedonia was among those who ate more than three meals, at 35.7%. The level of severe anxiety increased, which consisted of 4.3% coffee consumption with the level of anxiety, and the highest level of anxiety in mild anxiety who consumed tea almost never at 13.7%. It noted the highest normal anhedonia among those whose average tea consumption was sometimes 78.5%, while those whose average coffee consumption was often 74.1%.

**Table 3:** The associations between anhedonia and anxiety with food consumption.

Variables	Total	SHAPS		*p*-value	GAD-7				*p*-value
		Normal	Abnormal		Minimal	Mild	Moderate	Severe	
No. meals/day									
One meal	65 (16.5)	43 (66.2)	22 (33.8)	0.478	13 (3.3)	34 (8.7)	8 (2)	10 (2)	0.002
Two meals	232 (59)	168 (72.4)	64 (27.6)		54 (13.7)	108 (27.5)	42 (10.7)	28 (7.1)	
Three meals	82 (20.9)	63 (76.8)	19 (23.2)		38 (9.7)	32 (8.1)	10 (2.5)	2 (0.5)	
>3 meals	14 (3.6)	9 (64.3)	5 (35.7)		2 (0.5)	7 (1.8)	2 (0.5)	3 (0.8)	
Coffee (Americano, Cappuccino, etc.) consumption									
1–3 times/month	46 (11.7)	31 (67.4)	15 (32.6)	0.649	17 (4.3)	31 (7.9)	11 (2.8)	6 (1.5)	0.198
Once a weak	65 (16.5)	45 (69.2)	20 (30.8)		11 (2.8)	20 (5.1)	10 (2.5)	1 (0.3)	
2–4 times/week	42 (10.7)	28 (66.7)	14 (33.3)		21 (5.3)	51 (13)	12 (3.1)	15 (3.8)	
Once a day	99 (25.2)	78 (78.8)	21 (21.2)		7 (1.8)	15 (3.8)	8 (2)	6 (1.5)	
2–3 times/day	36 (9.2)	24 (66.7)	12 (33.3)		2 (0.5)	2 (0.5)	3 (0.8)	0.0 (0)	
4–5 times/day	7 (1.8)	5 (71.4)	2 (28.6)		34 (8.7)	45 (11.5)	11 (2.8)	8 (2)	
Never	98 (24.9)	72 (73.5)	26 (26.5)		17 (4.3)	31 (7.9)	11 (2.8)	6 (1.5)	
Saudi coffee consumption									
1–3 times/month	18 (4.6)	14 (77.8)	4 (22.2)	0.326	8 (2)	6 (1.5)	2 (0.5)	2 (0.5)	0.623
Once a weak	24 (6.1)	17 (70.8)	7 (29.2)		6 (1.5)	12 (3.1)	5 (1.3)	1 (0.3)	
2–4 times/week	50 (12.7)	32 (64)	18 (36)		15 (3.8)	20 (5.1)	10 (2.5)	5 (1.3)	
Once a day	155 (39.4)	115 (74.2)	40 (25.8)		41 (10.4)	77 (19.6)	25 (6.4)	12 (3.1)	
2–3 times/day	98 (24.9)	75 (76.5)	23 (23.5)		20 (5.1)	48 (12.2)	15 (3.8)	15 (3.8)	
4–5 times/day	12 (3.1)	9 (75)	3 (25)		3 (0.8)	4 (1)	2 (0.5)	3 (0.8)	
>5 times/day	8 (2)	6 (75)	2 (25)		4 (1)	2 (0.5)	1 (0.3)	1 (0.3)	
Never	28 (7.1)	15 (53)	13 (46.4)		10 (2.5)	12 (3.1)	2 (0.5)	4 (1)	
Tea consumption									
1–3 times/month	25 (6.4)	11 (44)	14 (56)	0.063	6 (1.5)	10 (2.5)	5 (1.3)	4 (1)	0.040
Once a weak	49 (12.5)	34 (69.4)	15 (30.6)		11 (2.8)	23 (5.9)	13 (3.3)	2 (0.5)	
2–4 times/week	69 (17.6)	51 (73.9)	18 (26.1)		20 (5.1)	31 (7.9)	11 (2.8)	7 (1.8)	
Once a day	103 (26.2)	81 (78.6)	22 (21.4)		30 (7.6)	48 (12.2)	18 (4.6)	7 (1.8)	
2–3 times/day	53 (13.5)	38 (71.7)	15 (28.3)		18 (4.6)	22 (5.6)	6 (1.5)	7 (1.8)	
4–5 times/day	10 (2.5)	8 (80)	2 (20)		4 (1)	5 (1.3)	0.0 (0)	1 (0.3)	
>5 times/day	7 (1.8)	4 (57.1)	3 (42.9)		2 (0.5)	1 (0.3)	0.0 (0)	4 (1)	
Never	77 (19.6)	56 (72.7)	21 (27.3)		16 (4.1)	41 (10.4)	9 (2.3)	11 (2.8)	
Fast-food consumption/week									
Once	186 (47.3)	138 (74.2)	48 (25.8)	0.219	43 (10.9)	91 (23.2)	28 (7.1)	24 (6.1)	0.002
Twice	71 (18.1)	56 (78.9)	15 (21.1)		13 (3.3)	42 (10.7)	13 (3.3)	3 (0.8)	
Three times	40 (10.2)	27 (67.5)	13 (32.5)		13 (3.3)	20 (5.1)	4 (1)	3 (0.8)	
<3 times	24 (6.1)	14 (58.3)	10 (41.7)		6 (1.5)	8 (2)	4 (1)	6 (1.5)	
Never	72 (18.3)	48 (66.7)	24 (33.3)		32 (8.1)	20 (5.1)	13 (3.3)	7 (1.8)	
Sugary drinks consumption/week									
Once	110 (28)	84 (76.4)	26 (23.6)	0.460	29 (7.4)	59 (15)	14 (3.6)	8 (2)	0.179
Twice	39 (9.9)	31 (79.5)	8 (20.5)		7 (1.8)	23 (5.9)	6 (1.5)	3 (0.8)	
Three times	37 (9.4)	25 (67.6)	12 (32.4)		11 (2.8)	16 (4.1)	7 (1.8)	3 (0.8)	
<3 times	33 (8.4)	24 (72.7)	9 (27.3)		11 (2.8)	10 (2.5)	4 (1)	8 (2)	
Never	174 (44.3)	119 (68.4)	55 (31.6)		49 (12.5)	73 (18.6)	31 (7.9)	21 (5.3)	
Fried food consumption/week									
Once	147 (37.4)	102 (69.4)	45 (30.6)	0.719	39 (9.9)	65 (16.5)	30 (7.6)	13 (3.3)	0.574
Twice	73 (18.6)	54 (74)	19 (26)		14 (3.6)	40 (10.2)	10 (2.5)	9 (2.3)	
Three times	40 (10.2)	30 (75)	10 (25)		11 (2.8)	19 (4.8)	6 (1.5)	4 (1)	
<3 times	25 (6.4)	16 (64)	9 (36)		6 (1.5)	12 (3.1)	3 (0.8)	4 (1)	
Never	108 (27.5)	81 (75)	27 (25)		37 (9.4)	45 (11.5)	13 (3.3)	13 (3.3)	
Pastry food consumption/week									
Once	183 (35.1)	101 (73.2)	37 (26.8)	0.353	38 (9.7)	67 (17)	27 (6.9)	6 (1.5)	0.173
Twice	83 (21.1)	55 (66.3)	28 (33.7)		20 (5.1)	37 (9.4)	16 (4.1)	10 (2.5)	
Three times	54 (13.7)	44 (81.5)	10 (18.5)		16 (4.1)	25 (6.4)	5 (1.3)	8 (2)	
<3 times	42 (10.7)	31 (73.8)	11 (26.2)		9 (2.3)	21 (5.3)	6 (1.5)	6 (1.5)	
Never	76 (19.3)	52 (68.4)	24 (31.6)		24 (6.1)	31 (7.9)	8 (2)	13 (3.3)	
Soft drinks consumption/week									
Once	114 (29)	86 (75.4)	28 (24.6)	0.836	26 (6.6)	60 (15.3)	21 (5.3)	7 (1.8)	0.070
Twice	49 (12.5)	36 (73.5)	13 (26.5)		6 (1.5)	25 (6.4)	12 (3.1)	6 (1.5)	
Three times	47 (12)	34 (72.3)	13 (27.7)		14 (3.6)	21 (5.3)	8 (2)	4 (1)	
<3 times	43 (10.9)	29 (67.4)	14 (32.6)		15 (3.8)	16 (4.1)	5 (1.3)	7 (1.8)	
Never	140 (35.6)	98 (70)	42 (30)		46 (12.7)	59 (15)	16 (4.1)	19 (4.8)	
Vegetables consumption/week									
Once	73 (18.6)	46 (63)	27 (37)	0.008	20 (5.1)	32 (8.1)	15 (3.8)	6 (1.5)	0.638
Twice	66 (16.8)	46 (69.7)	20 (30.3)		19 (4.8)	28 (7.1)	13 (3.3)	6 (1.5)	
Three times	111 (28.2)	83 (74.8)	28 (25.2)		29 (7.4)	53 (13.5)	14 (3.6)	15 (3.8)	
<3 times	116 (29.5)	94 (82)	22 (19)		36 (9.2)	54 (13.7)	14 (3.6)	12 (3.1)	
Never	27 (6.9)	14 (52.9)	13 (48.1)		3 (0.8)	14 (3.6)	6 (1.5)	4 (1)	
Fruit consumption/week									
Once	117 (29.8)	83 (70.9)	34 (29.1)	0.113	32 (8.1)	55 (14)	21 (5.3)	9 (2.3)	0.605
Twice	88 (22.4)	60 (68.2)	28 (31.8)		20 (5.1)	41 (10.4)	16 (4.1)	11 (2.8)	
Three times	71 (18.1)	57 (80.3)	14 (19.7)		18 (4.6)	36 (9.2)	8 (2)	9 (2.3)	
<3 times	48 (12.2)	39 (81.3)	9 (18.8)		20 (5.1)	18 (4.6)	5 (1.3)	5 (1.3)	
Never	69 (17.6)	44 (63.8)	25 (36.2)		17 (4.3)	31 (7.9)	12 (3.1)	9 (2.3)	
Water consumption/day									
2–3 cups	133 (33.8)	96 (72.2)	37 (27.8)	0.754	32 (8.1)	69 (17.6)	18 (4.6)	14 (3.6)	0.478
4–5 cups	152 (38.7)	112 (73.7)	40 (26.3)		42 (10.7)	71 (18.1)	22 (5.6)	17 (4.3)	
>5 cups	108 (27.5)	75 (69.4)	33 (30.6)		33 (8.4)	41 (10.4)	22 (5.6)	12 (3.1)	
Nutrition supplements intake									
No	175 (44.5)	127 (72.6)	48 (27.4)	0.932	50 (12.7)	80 (20.4)	26 (6.6)	19 (4.8)	0.617
Yes (regularly)	74 (18.8)	52 (70.3)	22 (29.7)		18 (4.6)	30 (7.6)	17 (4.3)	9 (2.3)	
Sometimes (irregularly)	144 (36.6)	104 (72.2)	40 (27.8)		39 (9.9)	71 (18.1)	19 (4.8)	15 (3.8)	

Data presented as *n* (%) for categorical variables; *p*-value < 0.05 considered significant.

Consuming fast food once a week increased the level of severe anxiety at the rate of 6.1% of the individuals in the sample. Non-consumers of fried and baked foods were equal at a level of extreme anxiety at 3.3%. Mild anxiety among soft drink consumers increased once a week at 15.3%. The highest rate of mild anxiety was among those who consumed vegetables more than three times a week, reaching 13.7%. The highest rate of mild anxiety was among those who consumed fruits once a week, reaching 14%. The highest rate of mild anxiety was among those who drank 4–5 cups, reaching 18.1%.

The results showed the highest percentage of abnormal anhedonia among those whose average consumption of sugary foods very often reached 33.1%. It found that the highest percentage of abnormal anhedonia was among those who consumed fast food and fried foods more than three times a week, where their percentage reached 41.7 and 36%, respectively, while the percentage among those who consumed sugary drinks three times a week was the highest, reaching 32.4%. The results also showed the highest percentage of natural anhedonia among those who ate pastry three times a week, reaching 81.5%. The highest percentage of abnormal anhedonia was among those who consumed soft drinks more than three times a week, reaching 32.6%. There is a moral significance between vegetable consumers and anhedonia at 0.008. The highest percentage of natural anhedonia was among those who consumed vegetables more than three times a week, reaching 82%. The highest percentage of natural anhedonia was among those who consumed vegetables more than three times a week, reaching 82%.

### 3.4 The associations between anhedonia and anxiety with health and lifestyle

Table 4. shows the relationship between health and lifestyle and anhedonia and anxiety. Non-smoker participants were the highest in the sample, with severe anxiety at 9.9, 42% with mild anxiety, 73.1% with normal anhedonia, and 26.9% suffering from abnormal anhedonia. However, regarding the relation of sleeping hours with the level of anxiety, it is morally significant at *p* 0.005. Anhedonia was abnormal among those who slept more than 7 h, the highest percentage at 28.3%. Coronavirus infectious, the highest level of anxiety was mild, at 25.4% for infected participants, and 20.6% for non-infected, with the highest percentage of abnormal anhedonia among those who had previously been infected with the coronavirus, at 29.6%.

**Table 4:** The associations between anhedonia and anxiety with health and lifestyle.

Variables	Total	SHAPS		*p*-value	GAD-7				*p*-value
		Normal	Abnormal		Minimal	Mild	Moderate	Severe	
Contraceptive									
Yes	49 (12.5)	39 (79.6)	10 (20.4)	0.186	14 (3.6)	25 (6.4)	7 (1.8)	3 (0.8)	0.021
No	267 (67.9)	194 (72.7)	73 (27.3)		60 (22.5)	126 (32.1)	47 (12)	34 (8.7)	
Not apply	77 (19.6)	50 (64.9)	27 (35.1)		33 (8.4)	30 (7.6)	8 (2)	6 (1.5)	
COVID-19 Infected									
Yes	204 (51.9)	150 (73.5)	54 (26.5)	0.486	55 (14)	100 (25.4)	28 (7.1)	21 (5.3)	0.549
No	189 (48.1)	133 (70.4)	56 (29.6)		52 (13.2)	81 (20.6)	34 (8.7)	22 (5.6)	
Smoke Status									
1–3/month	6 (1.5)	3 (50)	3 (50)	0.299	1 (0.3)	3 (0.8)	0.0 (0)	2 (0.5)	0.702
Once a month	1 (0.3)	0.0 (0)	1 (100)		0.0 (0)	1 (0.3)	0.0 (0)	0.0 (0)	
Once a week	1 (0.3)	1 (100)	0.0 (0)		1 (0.3)	0.0 (0)	0.0 (0)	0.0 (0)	
2–3 times a week	4 (1)	3 (75)	1 (25)		2 (0.5)	1 (0.3)	1 (0.3)	0.0 (0)	
Daily	20 (5.1)	12 (60)	8 (40)		6 (1.5)	11 (2.8)	1 (0.3)	2 (0.5)	
Non-smoking	361 (91.9)	264 (73.1)	97 (26.9)		97 (24.7)	165 (42)	60 (15.3)	39 (9.9)	
Sleeping hours/day									
<4 h	16 (4.1)	8 (50)	8 (50)	0.121	1 (0.3)	9 (2.3)	0.0 (0)	6 (1.5)	0.005
4–6 h	218 (55.5)	161 (73.9)	57 (26.1)		56 (14.2)	105 (26.7)	34 (8.7)	23 (5.9)	
≧7 h	159 (40.5)	114 (71.7)	45 (28.3)		50 (12.7)	67 (17)	28 (7.1)	14 (3.6)	

Data presented as *n* (%) for categorical variables; *p*-value < 0.05 considered significant.

**Figure 1:**
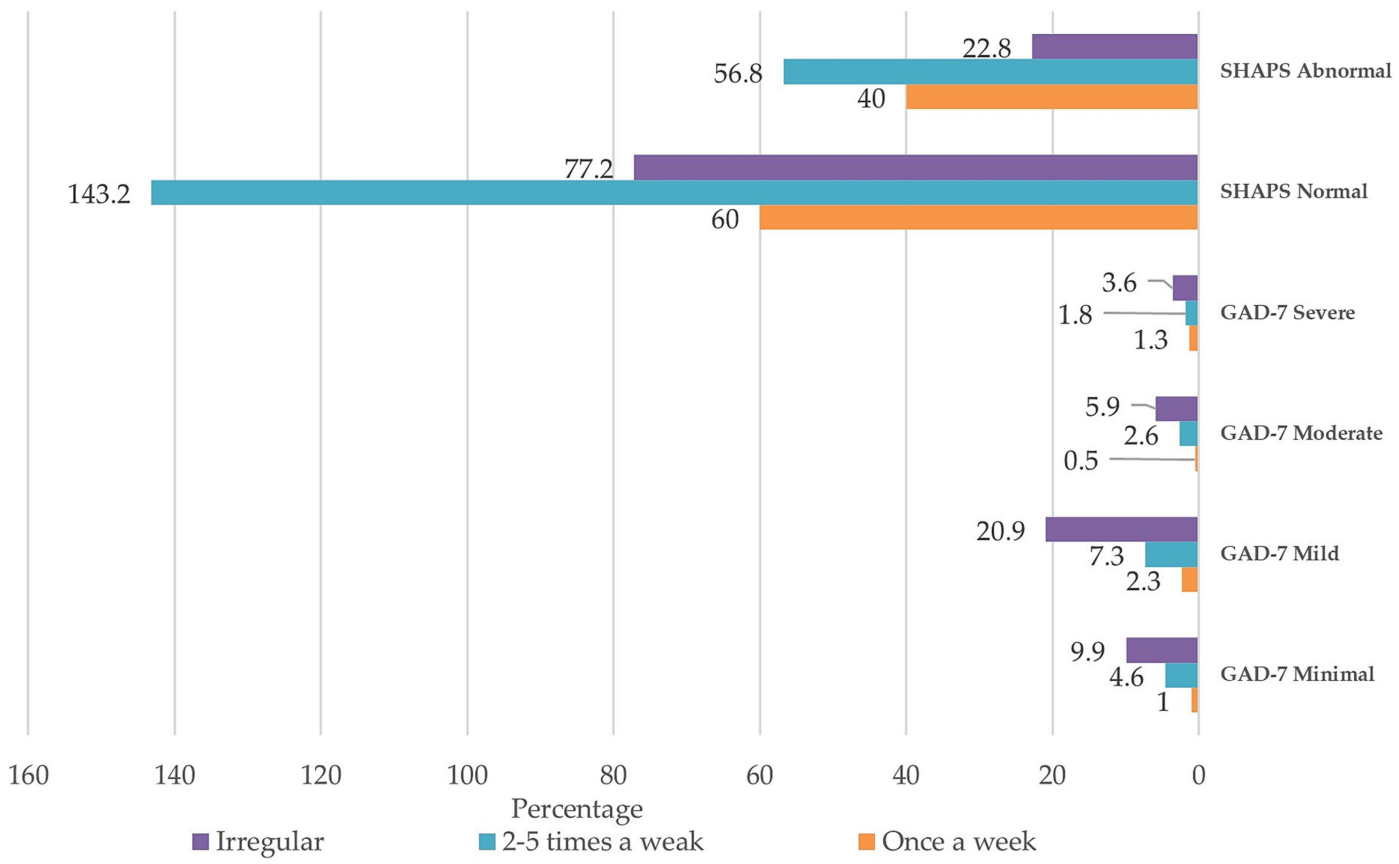
The frequency of physical activity practice is according to anhedonia and anxiety.

Figure 1 shows the frequency of physical activity practice according to anhedonia and anxiety—participants who do physical activity two to five times a week have about 56.8% abnormal anhedonia, while in normal anhedonia, it is 143.2%. About 7% of participants in the study who had mild anxiety do physical activity two to five times a week, while 2.6 and 1.8% had moderated and severe anxiety. Irregular physical activity was higher in all classifications of anxiety. There was no significant result between physical activities and anhedonia and anxiety.

### 3.5 Mean body mass index and weight with anhedonia scale and anxiety

Table 5 shows the results of mean body weight by anxiety and anhedonia scales. It was found that the highest category of participants was in the category of mild anxiety, with a mean body mass index of 25.71 and a mean weight of 67.09 kg. Also, it was found that the highest category of participants was normal anhedonia, with a mean BMI of 25.45 and a mean weight of 66.48 kg.

**Table 5:** Mean BMI and weight with anhedonia and anxiety.

Variables		SHAPS		*p*-value	GAD-7				*p*-value
		Normal	Abnormal		Minimal	Mild	Moderate	Severe	
	n	283	110		107	181	62	43	
BMI	Mean ± (SD)	25.45 ± 4.67	25.39 ± 4.93	0.880	24.84 ± 4.43	25.71 ± 4.78	25.57 ± 5.45	25.51 ± 4.242	0.929
Weight		66.5 ± 13.85	66.95 ± 16.16	0.279	66.46 ± 14.27	67.09 ± 14.39	66.19 ± 16.79	65.63 ± 12.35	0.505

Mean ± (SD) Standard Deviation; there is no significant difference *p* > 0.05.

### 3.6 The liner regression analyses

The linear regression analysis associated with the dependent variable was the number of meals per day with variables as shown in Table 6. There was a significant association between age and the main number of meals per day—also, the consumption of fruit and drinking water was significant in linear regression. There was no significance between the number of meals per day and anhedonia; however, the main number of meals per day was statistically significant with the total GAD-7 scale score.

**Table 6:** The regression analyses.

Variables	B ± SE	Std. Beta	*p*-value	95.0% confidence interval for B	
				Lower bound	Upper bound
Age	0.207 ± 0.082	0.130	0.012	0.045	0.369
BMI classification	0.028 ± 0.064	0.108	0.660	−0.097	0.154
Consumption of fast-food	0.010 ± 0.026	0.023	0.685	−0.040	0.061
Consumption of vegetables	0.029 ± 0.031	0.049	0.350	−0.032	0.089
Consumption of fruits	−0.055 ± 0.025	−0.112	0.032	−0.105	−0.005
Drinking water	0.098 ± 0.046	0.108	0.034	0.008	0.189
Total score SHAPS	−0.009 ± 0.024	−0.030	0.721	−0.057	0.039
Total score GAD-7	−0.086 ± 0.041	−0.112	0.039	−0.167	−0.004

Data presented as BSE from linear regression. The dependent variable was the number of meals/day. *p* < 0.05 was considered statistically significant.

## 4 Discussion

This, to our knowledge, is the first study of the prevalence of anhedonia, anxiety, and the associated food consumed in Saudi Arabia, especially among postgraduates at Qassim University students. The study is consistent with that of AlShamlan and Abdelreheem (38, 39), who concluded that the prevalence of anxiety and depression was highest among medical school students. Additionally, the prevalence of the condition was found to be higher among females. Baldassin’s study results are similar to this study, which reported high levels of depression and anxiety among second and fourth-academic-level students (40). Students in the study phase were suffering from levels of anxiety and depression (41). Students in this current study suffered from anxiety during the research phase. A study that was conducted in the United Kingdom found similar results to the current research, and it found a relation between consuming unhealthy foods and higher depression symptoms, as well as a relation between consuming ‘healthy’ foods and lower depression symptoms among students (42).

This study’s results showed significant similarities in the relationship between food consumption and depression (43). The study results confirm the number of daily meals participants consume, two meals per day, similar to AL-Rethaiaa’s study results (44). Severe depression is associated with unhealthy dietary intake, worse dietary quality, and higher intake of sweet food, fast food and savory snacks. Depressive symptoms were positively associated with sugary drink intake. On the contrary, depressive symptoms are not associated with fruits and vegetables, which are similar to the results of this study (45). This study found that individuals with no depressive levels consumed more supplements and healthy foods. The most important results of the study showed a strong positive association between fruits and vegetables but a negative association with sugary drinks, fast food, and fried foods (46), pizza (47), baked goods consumption (48) and sweets and pastries (49).

The study results agree that depressive symptoms are associated with increased consumption of total energy and energy from sweet snacks (50). The results of the study agree with the theory that high consumption of fruits (51) and vegetables is associated with lower levels of depression and anxiety (52). The study’s results confirmed an inverse relationship between vegetables, fruit consumption, and depression (53, 54); indications were related to less consumption of fruits and more fast food or canned food and soft drinks among female college students in the United Arab Emirates (55). In a study conducted on 22,817 participants, including 4,743 people with depression, the result showed that tea consumption is correlated with a lower risk of depression (56). Green tea consumption was related to lower depression symptoms (57). Navarro et al. studied 14,413 university graduates of the ‘Seguimiento Universidad de Navarra (SUN) cohort. Participants who drank at least four cups of coffee per day showed a significantly lower risk of depression than participants who drank less than one cup of coffee per day (58). More prominent adherence to the Mediterranean diet and day-by-day tea drinking appears to have a helpful impact on depressive side effects (59). In a study conducted on women over a 10-year follow-up, they found the risk of depression decreased with increased consumption of caffeinated coffee (60). The study’s results by Park and Moon supported the protective effect of coffee on the risk of depression (61). Caffeine consumption over 68 mg/day reduces depression risk (62), while natural products like fish, walnuts, coffee, tea, dietary fiber, vitamin B_6_, and greasy acids protect against anxiety and reduce depression risk (63).

Research shows that diet is linked to depression symptoms, with increased cravings for carbohydrates and fatty foods during depressive episodes (64). Moderate carbohydrate and high protein consumption are associated with a lower chance of depression (65). Vegetarian diets may protect against depression symptoms (66, 67). However, those who do not consume meat have a higher risk of depression and anxiety (68). The association between depressive symptoms and vegetarian nutrition may be due to iron, zinc, vitamin D, vitamin B_12_, and calcium deficiencies (69). This study explores the relationship between anhedonic features, mental health, and food pleasure in individuals with anhedonia (70). It provides an account of factors affecting enjoyment from eating and meals and explores the potential effects of varying degrees of anhedonia on eating patterns and food choices (20). Understanding the experiences of anhedonic people is crucial for supporting and easing their condition in the future.

Research has shown a correlation between depression and physical activity, with higher levels of physical activity leading to lower depression levels (71). Additionally, team sports participation has been linked to reduced depression and anxiety levels among college students (72). Psychophysiology examines how stress, anxiety, and depression affect athletes’ physical abilities. Regular physical activity lowers symptoms of depression and anxiety. Coping mechanisms that work enhance health results (73). The relationship between psychology and physiology in sports; psychophysiology shows how psychological variables impact physiological reactions. Athletes can better control their physiological reactions with techniques like biofeedback and mental training, improving their performance and fostering overall athlete development (74).

However, non-smokers are more anxious than smokers, contradicting previous studies that found higher rates of depressive symptoms and anxiety among college students (75). Smoking is often a result of pleasure and anxiety relief, and medical students may smoke due to psychological stress related to their academics (76). Sleep quality is also linked to depression and anxiety, with poor sleep quality strongly associated with higher anxiety levels (77). This contradicts previous research suggesting that anxiety symptoms, sleep problems, and higher perceived stress among college students increased during the COVID-19 outbreak (78). The study found that 0.9% of respondents experienced severe anxiety, 2.7% moderate anxiety, and 21.3% mild anxiety (79).

The study reveals that students with financial burdens and living alone experience high levels of anxiety and depression symptoms (80). This contradicts previous research suggesting that students with high life satisfaction are more likely to be urban and semi-urban (81). The lifetime prevalence of anxiety and depression is higher in urban areas. In Colombia, poor financial conditions and dispersed areas have lower mental health problems (82). The study also found that living density is associated with higher anxiety and stress, and income poverty is linked to increased anxiety and stress (67). Prevalence of anxiety and depression among university students with body dysmorphic disorder, highlighting the importance of addressing mental health concerns in this population (83). The study reveals that college students with a higher average BMI index experience higher rates of depression compared to those with a lower BMI index (84).

The study’s limitations include the cross-sectional design, lack of longitudinal data, self-reported data, and diversity in non-clinical settings. It is possible that the study did not consider all the participant’s dietary complexity. The strength study focuses on university students who are at high risk for eating disorders and related health issues. It collects comprehensive data through validated questionnaires. The study has a large sample size of 393 participants, which ensures reliable statistical analyses. The study follows ethical guidelines and identifies significant predictors of eating consumption, such as fat and sugar consumption. It adds to the existing literature on eating disorders in Saudi Arabia while also raising awareness of the health implications for university students. These strengths provide valuable insights into eating patterns among university students. The study on anxiety-related eating consumption among Qassim University students has limitations and makes several recommendations for future research. These include using a longitudinal design to track changes in eating behaviors over time, including diverse populations, incorporating psychological factors, developing and testing interventions to reduce emotional eating, incorporating qualitative research to understand personal experiences and motivations, exploring social influences on eating consumption, assessing dietary quality, and exploring the cultural context in different regions of Saudi Arabia and the Gulf countries. These recommendations aim to give university students a better understanding of eating patterns and its health consequences.

## 5 Conclusion

The consumption of fat, sugary containing foods by students is resulting in an elevated risk of metabolic diseases, such as diabetes and heart disease, as well as obesity and eating disorders. Participants’ enthusiasm, fat, and sugary intake substantially influenced their eating habits. The results underscore the significance of comprehending consumption eating in order to comprehend the lifestyle and dietary patterns of Saudi Arabia. The study underlines the necessity for interventions that promote healthy eating habits and manage eating in order to enhance health outcomes and mitigate risks for university students. The research provides valuable data to the existing literature on consumption eating and dietary patterns, with a particular emphasis on Saudi Arabia. The study concludes that university students have a substantial concern regarding eating and additional research is required to encourage healthier lifestyles. Further investigation is needed to understand the complex interplay between anhedonia, mental health, and food pleasure.

## Data Availability

The raw data supporting the conclusions of this article will be made available by the authors without undue reservation.
